# *Curcuma deliniana*, a Rare New *Curcuma* Species (Zingiberaceae) Previously Misidentified as *C. wenyujin*

**DOI:** 10.1002/ece3.74307

**Published:** 2026-09-16

**Authors:** Juan Chen, Hui‐Hong Wang, En‐Wei Tian, Nian‐He Xia

**Affiliations:** ^1^ State Key Laboratory of Plant Diversity and Specialty Crops/Key Laboratory of National Forestry and Grassland Administration on Plant Conservation and Utilization in Southern China/Guangdong Provincial Key Laboratory of Digital Botanical Garden, South China Botanical Garden Chinese Academy of Sciences Guangzhou China; ^2^ South China National Botanical Garden Guangzhou China; ^3^ School of Traditional Chinese Medicine Southern Medical University Guangzhou China

**Keywords:** *Curcuma wenyujin*, new species, plastomes, single‐copy orthologous genes, taxonomy

## Abstract

A new triploid *Curcuma* species from Guangxi in China, *C. deliniana*, is described and illustrated. It was previously misidentified as the valuable medicinal plant *C. wenyujin* but can be readily distinguished from the latter by its rarely branched rhizomes, slender shoots, narrow leaf blades, and small inflorescences. The species is used as a substitute or adulterant of another medicinal species, *C. kwangsiensis* , from which it differs in larger, nearly globose main rhizomes; green leaf blades that are glabrous adaxially and sparsely puberulent or glabrous abaxially; inflorescence with fewer, loosely arranged bracts; more broadly elliptic coma bracts, non‐reflexed at tips; nearly rounded, whitish green fertile bracts with purple tips; white corolla lobes; and chromosome number 2*n* = 63. Phylogenetic analyses based on the plastid genomes recovered it as sister to *C.* aff. *plicata* while analyses based on single‐copy orthologous genes placed it as sister to *C. kwangsiensis* with very weak support. Due to its limited populations and potential risk of continuing decline in the number of mature individuals, it is provisionally assessed as Endangered (EN) according to the IUCN Red List Guidelines. This discovery not only provides important insights into the species identification and evolutionary relationships within the economically important genus *Curcuma* but also highlights the importance of ensuring the authenticity of herbal raw materials.

## Graphical abstract

A new triplod *Curcuma* species from Guangxi in China, *C. deliniana*, is described and illustrated. It was previously misidentified as the medicinal plant *C. wenyujin* and used as a substitute or adulterant of *C. kwangsiensis* , whereas it differs from the latter two species. Phylogenetic and morphological analyses confirm its status as a distinct new species. Meanwhile, its conservation status is provisionally assessed as Endangered (En).

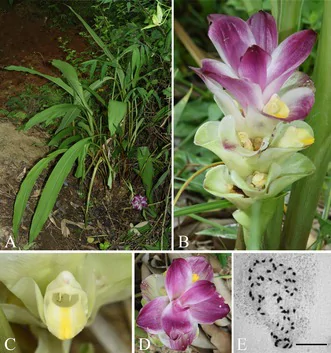

## 1 Introduction

*Curcuma* L. (Zingiberaceae) is an economically important genus, mainly distributed in South and Southeast Asia with many species extending to China, Australia, and the South Pacific. Recent estimates of its total species number vary from 50 (Larsen et al. 1998; Wu and Larsen 2000) to 120 (Leong‐Škorničková et al. 2007) or more than 150 (Leong‐Škorničková et al. 2023). Numerous new *Curcuma* species have been described in recent years (Chen et al. 2015, 2019, 2021; Luu et al. 2017; Soonthornkalump et al. 2020, 2021, 2022; Saensouk et al. 2021, 2024; Soonthornkalump 2024; Phan et al. 2025; Pham et al. 2026), continuously increasing its total number. Meanwhile, the most recent phylogenetic study on the genus (Záveská et al. 2012) recognized three subgenera mainly based on spur types: subg. *Curcuma*, subg. *Hitcheniopsis* (Baker) K. Schum., and subg. *Ecomatae* Škorničková & Šída. Despite ongoing research, unresolved taxonomic issues still hinder a satisfactory taxonomic treatment of the genus and the identification and classification of *Curcuma* species are challenging tasks for botanists, as discussed in Leong‐Škorničková et al. (2008) and Chen et al. (2021). Specifically, the application of the names of many *Curcuma* species described in early taxonomic literature remains ambiguous. Numerous synonyms, especially those established solely based on herbarium specimens, are unreliable and require critical reconsideration. Furthermore, differing levels of variation within *Curcuma* species have triggered a longstanding controversy concerning species concepts and the boundaries between taxa. Some seed‐setting species (2*n* = 42, 84) exhibit a wide range of intraspecific variability, while others (2*n* = 63) that reproduce vegetatively by rhizomes have limited variation (which, although superficially quite similar, possess subtle but stable differences) (Leong‐Škorničková et al. 2010). Recently, more molecular data such as AFLP markers (Záveská et al. 2011), plastomes (Gui et al. 2020), and targeted low‐copy nuclear genes (Skopalíková et al. 2023) have been used to investigate the phylogenetic relationships among *Curcuma* species. Among these, plastomes and single‐copy nuclear genes are widely used for species identification rapidly increasing in popularity because they are relatively fast to process and offer large datasets of discrete characters.

During the revision of *Curcuma* from China, several specimens (*X. X. Chen 78914* & *78912*) attracted our attention. These specimens were collected from cultivated plants at the Guangxi Medicinal Botanical Garden, which were originally introduced from Heng County (now Hengzhou City) in Guangxi. They are characterized by rarely branched rhizomes, narrow leaf blades that are glabrous adaxially and puberulent abaxially, and lateral inflorescences. Initially, Chen (1980) identified them as *C. elata* Roxb., possibly due to their large rhizomes. Later, Liu and Wu (1999) treated them as *C. wenyujin* Y. H. Chen & C. Ling and this treatment was accepted by Wu and Larsen (2000) in *Flora of China*. The identification of these specimens within the genus is questionable because the color of the bracts and flower parts are important characters for species determination which are unseen in herbarium specimens. Thus, living material including flowers and rhizomes is crucial for an accurate understanding their identities. Fortunately, during our field work in Heng County in 2020, an interesting *Curcuma* species (belonging to subg. *Curcuma*) with large, rarely branched rhizomes, narrow leaf blades, and lateral inflorescences with purplish red coma bracts was collected, matching well with *C. elata* sensu X. X. Chen. Meanwhile, its rhizomes were successfully transplanted to South China Botanical Garden. Although this plant has large rhizomes similar in size to those of *C. elata* or *C. wenyujin*, its rhizome shape more resembles that of *C. kwangsiensis* S. G. Lee & C. F. Liang. To determine which species it is closely allied to, phylogenetic analyses using chloroplast genomes and single‐copy orthologous genes as well as morphological analysis were conducted. After detailed studies, we concluded that the material from Guangxi represents a new species which is described and illustrated herein.

## 2 Materials and Methods

### 2.1 Morphological Analysis

Names of taxa currently recognized as members of *Curcuma* from China have been investigated, as well as several other names from adjacent countries. Types and original materials including herbarium sheets, drawings/icons and manuscripts were searched and checked. All *Curcuma* specimens in the following Chinese herbaria were examined: GXMG, GXMI, HITBC, IBK, IBSC, IMD, IMDY, KUN and PE; additional digitized materials from China and neighboring countries (Laos and Vietnam) in the following herbaria have also been studied through GBIF (https://www.gbif.org/) and Global plants on JSTOR (https://plants.jstor.org/).

The description of this new species was based on living plants cultivated at South China Botanical Garden. Quantitative traits (e.g., plant height, leaf size, and floral organ dimensions) were measured using a steel ruler (0.5 mm precision). The style of description and methods followed Chen et al. (2021) and the terminology in general followed Beentje (2016).

Chromosome counts were conducted on somatic metaphases using the root tip squash technique outlined in Chen et al. (2021). Actively growing root tips of cultivated plants which were introduced from the field were used to count the chromosome numbers. The number of chromosomes was determined from five complete well‐spread mitotic metaphase plates.

### 2.2 DNA Extraction, Sequencing, and Phylogenetic Reconstruction

DNA was extracted from silica gel‐dried leaf fragments. DNA extraction, quality assessment, library construction and paired‐end sequencing were conducted by Novogene Co. Ltd. (Beijing, China) using the Illumina PE150 platform. The Agencourt SPRIselect kit (Beckman Coulter, USA) was used to purify libraries and size‐select fragments of appropriate length. The initial libraries were PCR‐amplified to obtain sufficient material for sequencing. Library effective concentration (1.5 nM) was accurately quantified via quantitative PCR (qPCR) to ensure library quality. Approximately 20 Gb of raw sequencing data per sample were generated, except *C. exigua* N. Liu with 100 Gb raw data. Raw reads were quality‐trimmed with fastp to generate clean reads which were utilized for chloroplast genome assembly and annotation, as well as to identify and extract nuclear single‐copy orthologs.

For chloroplast genome analyses, 19 complete cp genomes representing 19 *Curcuma* species mainly from China were newly sequenced and assembled using GetOrganelle v.1.7.6.1 (Jin et al. 2020) (Table 1). Subsequently, these genomes were annotated using Geneious v.2019.0.3 (Kearse et al. 2012). A maximum likelihood (ML) phylogenetic tree was constructed using the TVM + F + R2 model with 1000 bootstrap replicates by IQ‐TREE v2.3.6 (Minh et al. 2020), with *C. alismatifolia* Gagnep. and *C. woodii* N.H.Xia & Juan Chen as outgroups.

**TABLE 1:** 19 *Curcuma* species used for plastid genome and single‐copy orthologous gene (SOG) phylogenetic analyses in this study, with localities, voucher information and GenBank accession numbers.

Species name	Locality	Voucher	Herbarium	GenBank accession no. (cpDNA)
*Curcuma* aff. *amarissima*	Cultivated at SCBG, Guangdong, China	*J. Chen 1714*	IBSC	PX957515
*C*. aff. *aromatica*	Cultivated at SCBG, Guangdong, China	*J. Chen CJ202407*	IBSC	PX957516
*C*. aff. *elata*	Enping, Guangdong, China	*J. Chen 202023*	IBSC	PX957517
*C*. aff. *plicata*	Simao, Yunnan, China	*J. Chen & H. H. Wang JChen032*	IBSC	PX957518
*C. alismatifolia*	Cultivated at SCBG, Guangdong, China	*J. Chen 201304*	IBSC	PQ490438
*C. deliniana*	Cultivated at SCBG, Guangdong, China	*J. Chen 202105*	IBSC	PX957519
*C. exigua*	Cultivated at SCBG, Guangdong, China	*J. Chen 202307*	IBSC	PQ490437
*C. gulinqingensis*	Wenshan, Yunnan, China	*J. Chen & H. H. Wang JChen136*	IBSC	PX957520
*C. kwangsiensis*	Hengzhou, Guangxi, China	*J. Chen et al. 060913*	IBSC	PZ512962
*C. longa*	Chongzuo, Guangxi, China	*J. Chen & H. H. Wang JChen099*	IBSC	PX957522
*C. nankunshanensis*	Cultivated at SCBG, Guangdong, China	*J. Chen 20063004*	IBSC	PQ490430
*C. phaeocaulis*	Wenshan, Yunnan, China	*J. Chen & K. Xu JChen182*	IBSC	PX957523
*C. rubescens*	Cultivated at SCBG, Guangdong, China	*J. Chen 20063002*	IBSC	PQ490441
*C. rubrobracteata*	Ximeng, Yunnan, China	*J. Chen et al. 17081601*	IBSC	PQ490440
*C. ruiliensis*	Ruili, Yunnan, China	*J. Chen 201927*	IBSC	PX957524
*C. wenyujin*	Cultivated at SCBG, Guangdong, China	*J. Chen 202303*	IBSC	PX957525
*C. woodii*	Cultivated at SCBG, Guangdong, China	*J. Chen & T. Wood 201010*	IBSC	PQ490435
*C. yingdeensis*	Yingde, Guangdong, China	*J. Chen et al. 17051801*	IBSC	PQ490436
*C. zanthorrhiza*	Cultivated at SCBG, Guangdong, China	*J. Chen 22052606*	IBSC	PQ490442

For nuclear genomic analyses, the genomes of *C. alismatifolia* and *C. petiolata* Roxb. were downloaded (Liao et al. 2024) and analyzed to identify single‐copy orthologs shared by both species using OrthoFinder v.2.5.4 (Emms and Kelly 2019), which served as a reference gene set. Nuclear sequences from all 19 samples were then assembled against this reference set using HybPiper v.2.3.3 (Johnson et al. 2016), followed by data quality verification. The assembled sequences were aligned using MAFFT v.7.525 (Katoh and Standley 2013). Potential paralogs were detected and removed by HybPiper v.2.3.3. Genes missing from any of the 19 samples or had an aligned length shorter than 1500 bp were filtered out to yield a final set of putative single‐copy orthologs. Individual gene trees were constructed for these orthologs using IQ‐TREE v2.3.6 under the ML framework. Branches with bootstrap support values below 50% were collapsed. Finally, a species tree was inferred from the resulting set of gene trees using ASTRAL v.5.7.3 (Zhang et al. 2018).

## 3 Results and Discussion

### 3.1 Phylogenetic Position of the New Species

**FIGURE 1:**
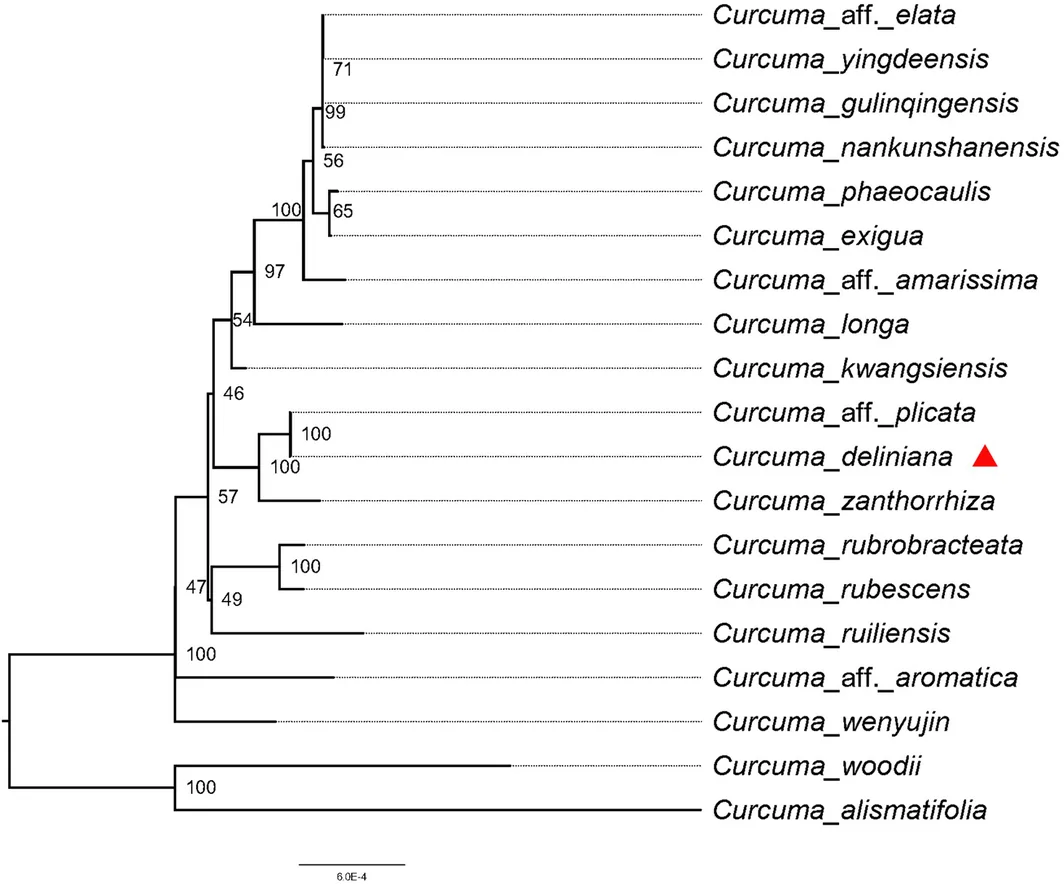
Maximum likelihood (ML) phylogenetic tree reconstructed from 19 complete plastid genomes from 19 *Curcuma* species. Numbers beside nodes indicate bootstrap support (BS) values from ML analysis. The new species described here is marked with a red triangle. Scale bar represents the number of nucleotide substitutions per site.

The 19 plastomes assembled in this study shared highly similar lengths and structures, with genome sizes ranging from 162,143 to 163,645 bp. These sizes are consistent with those previously documented for other *Curcuma* species (Liang and Chen 2021). All assembled genomes exhibited a typical quadripartite structure, consisting of one large single‐copy (LSC), one small single‐copy (SSC), and two inverted repeat (IRa and IRb) regions. The average overall GC content among the sequenced plastomes varied from 36.1% to 36.2%. The 19 chloroplast genomes contained 133–136 genes, including 83–87 protein‐coding genes, 38–40 transfer RNA (tRNA) genes and 8–10 ribosomal RNA (rRNA) genes. Additional sequence characteristics are summarized in Table 2. The plastome phylogeny revealed an unexpected finding that resolved the new species as sister to *C*. aff. *plicata* with strong support (BS = 100%; Figure 1), despite their significant morphological differences beyond their shared characters of pale yellow to creamy rhizomes. The new species can be readily distinguished from *C*. aff. *plicata* by the following morphological features: its shoots are slender and clumps are loose (vs. robust shoots and dense clumps); its main rhizome is broadly ovoid to globose (vs. narrowly ovoid); its leaf blades are spreading (vs. much erect); its coma bracts are purplish red (vs. pink); its fertile bracts are broadly ovate to rounded, loosely arranged (vs. narrowly ovate, densely arranged). Additionally, the new species is currently known only from southern Guangxi, whereas *C*. aff. *plicata* has been discovered in Ximeng, Menghai, Menglian Counties of southern Yunnan.

**TABLE 2:** Characteristics of 19 complete plastomes from 19 *Curcuma* species.

Species name	Total size (bp)	Size (bp)			GC content (%) (LSC/SSC/IR)	No. of genes (PCGs/tRNA/rRNA)
		Large single‐copy region (LSC)	Small single‐copy region (SSC)	Inverted repeats (IRs)		
*Curcuma* aff. *amarissima*	162,155	86,978	15,679	59,498	36.2 (34.0, 29.7, 41.2)	133 (87, 38, 8)
*C*. aff. *aromatica*	162,266	87,042	15,710	59,514	36.2 (34.0, 29.6, 41.1)	133 (87, 38, 8)
*C*. aff. *elata*	162,395	87,188	15,709	59,498	36.2 (34.0, 29.6, 41.2)	133 (87, 38, 8)
*C*. aff. *plicata*	162,349	87,142	15,707	59,500	36.2 (34.0, 29.7, 41.2)	133 (87, 38, 8)
*C. alismatifolia*	163,645	87,909	15,512	60,224	36.1 (33.9, 29.7, 40.9)	133 (83, 40, 10)
*C. deliniana*	162,289	87,089	15,700	59,500	36.2 (34.0, 29.7, 41.2)	133 (87, 38, 8)
*C. exigua*	162,187	86,995	15,694	59,498	36.2 (34.0, 29.7, 41.2)	136 (86, 40, 10)
*C. gulinqingensis*	162,221	87,039	15,684	59,498	36.2 (34.0, 29.7, 41.2)	133 (87, 38, 8)
*C. kwangsiensis*	162,290	87,112	15,678	59,500	36.2 (33.9, 29.7, 41.1)	136 (86, 40, 10)
*C. longa*	162,317	87,024	15,789	59,504	36.2 (34.0, 29.5, 41.1)	133 (87, 38, 8)
*C. nankunshanensis*	162,221	87,039	15,684	59,498	36.2 (34.0, 29.7, 41.2)	136 (86, 40, 10)
*C. phaeocaulis*	162,249	87,121	15,630	59,498	36.2 (34.0, 29.8, 41.1)	133 (87, 38, 8)
*C. rubescens*	162,256	87,031	15,733	59,492	36.2 (34.0, 29.6, 41.1)	134 (84, 40, 10)
*C. rubrobracteata*	162,143	86,932	15,731	59,480	36.2 (34.0, 29.6, 41.1)	134 (84, 40, 10)
*C. ruiliensis*	162,460	87,176	15,800	59,484	36.2 (33.9, 29.5, 41.1)	133 (87, 38, 8)
*C. wenyujin*	162,188	87,023	15,665	59,500	36.2 (34.0, 29.7, 41.1)	133 (87, 38, 8)
*C. woodii*	163,319	87,898	15,777	59,644	36.1 (33.9, 29.6, 41.1)	135 (85, 40, 10)
*C. yingdeensis*	162,221	87,039	15,684	59,498	36.2 (34.0, 29.7, 41.2)	136 (86, 40, 10)
*C. zanthorrhiza*	162,152	86,981	15,739	59,432	36.2 (34.0, 29.6, 41.1)	133 (83, 40, 10)

**FIGURE 2:**
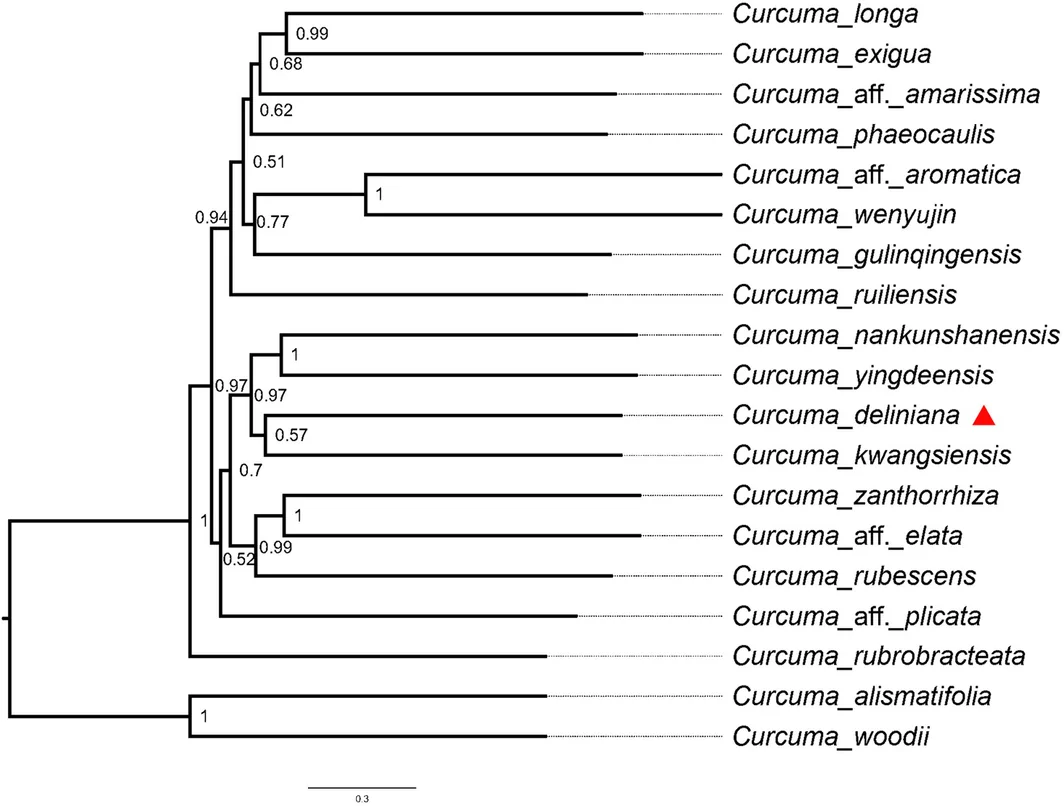
Species tree based on 973 single‐copy orthologous genes of 19 *Curcuma* species analyzed with ASTRAL. Numbers beside nodes indicate local posterior probabilities (PP). The new species described here is marked with a red triangle. The scale bar for internal branches indicates branch lengths in coalescent units. Branch lengths for terminal branches are arbitrary and are not estimated by ASTRAL.

Conflicting topologies were recovered from the single‐copy orthologous gene dataset. The raw sequencing data from 19 *Curcuma* samples yielded an average of 84.13 million reads per sample after quality filtering and removal of duplicates. Orthologous analysis of *C. alismatifolia* and *C. petiolata* genomes identified 5345 single‐copy orthologs shared by both species; these orthologs were used as the reference gene set. Based on the reference gene set, 973 single‐copy orthologous genes (SOGs) were consistently recovered across all samples and passed quality verification. Individual sample statistics of 973 genes including SOG alignment length and parsimony informative sites are presented in Table 3. Intriguingly, the species tree inferred from SOGs using the coalescence‐based method in ASTRAL revealed that the new species formed a poorly supported clade with *C. kwangsiensis* (PP = 0.57; Figure 2), which was further recovered as sister to another clade comprising *C. nankunshanensis* N. Liu, X. B. Ye & Juan Chen and *C. yingdeensis* N. H. Xia & Juan Chen, whereas it was distantly related to *C*. aff. *plicata* (Figure 2). Cytonuclear phylogenetic discordance among closely related taxa commonly arises from multiple evolutionary factors (reviewed in Larson et al. 2026). Incomplete lineage sorting and hybridization/introgression are recognized as prominent factors contributing to cytonuclear discordance across various taxa (Rieseberg and Soltis 1991; Morales‐Briones et al. 2018; McLay et al. 2023; Xu et al. 2025). Additionally, nuclear genomes and chloroplast genomes possess different evolutionary rates, inheritance modes and selective pressures, thus recording distinct evolutionary histories and eventually leading to inconsistent topological relationships in phylogenetic trees (Rieseberg and Soltis 1991; Maddison 1997; Lee‐Yaw et al. 2018; Xu et al. 2025). In this study, we primarily interpreted interspecific relationships within *Curcuma* based on nuclear phylogeny and overall phenotypic similarity. Plastid genomes are maternally inherited and susceptible to chloroplast capture triggered by hybridization and backcrossing, which may cause plastid phylogenies to fail to reflect true species relationships. Considering that ancient hybridization and polyploidization have been well documented as key evolutionary forces driving *Curcuma* diversification (Leong‐Škorničková et al. 2007; Záveská et al. 2012, 2016; Skopalíková et al. 2023), historical introgression represents a possible explanation for the cytonuclear topological conflicts observed in our analyses. Further analyses including expanded geographic and phylogenetic sampling and multiple analytical approaches are required to explore the exact causes as well as provide comprehensive insights into the evolutionary history of *Curcuma*.

**TABLE 3:** Summary statistics of 19 *Curcuma* genomes. Statistics include the number of clean reads, site completeness, number and percentage of genes recovered, proportion of missing data, SOG alignment length, and parsimony informative sites.

Species name	Clean reads	Site completeness	Genes recovered	Recovery rate (%)	Missing data (%)	SOG alignment length (bp)	Parsimony informative sites
*Curcuma* aff. *amarissima*	70,336,290	0.95	5282	98.82	5.45	7,075,311	3,595,897
*C*. aff. *aromatica*	70,059,539	0.96	5274	98.67	4.25	7,064,430	3,630,530
*C*. aff. *elata*	80,154,195	0.97	5281	98.80	3.32	7,071,993	3,675,033
*C*. aff. *plicata*	89,790,956	0.97	5281	98.80	3.46	7,070,937	3,666,671
*C. alismatifolia*	71,567,729	0.87	5061	94.69	12.54	6,892,071	3,219,632
*C. deliniana*	68,359,359	0.94	5260	98.41	6.13	7,057,293	3,562,170
*C. exigua*	336,486,466	0.99	5114	95.68	0.98	6,810,747	3,574,666
*C. gulinqingensis*	70,263,534	0.93	5202	97.32	7.34	7,004,745	3,479,543
*C. kwangsiensis*	69,119,156	0.94	5268	98.56	5.77	7,080,108	3,592,793
*C. longa*	69,446,183	0.95	5277	98.73	5.02	7,074,819	3,610,849
*C. nankunshanensis*	67,179,873	0.94	5264	98.48	5.66	7,065,687	3,579,457
*C. phaeocaulis*	70,698,616	0.91	5102	95.45	9.10	6,879,582	3,337,807
*C. rubescens*	69,491,502	0.94	5259	98.39	6.22	7,062,234	3,551,605
*C. rubrobracteata*	72,684,933	0.94	5250	98.22	6.45	7,054,593	3,546,750
*C. ruiliensis*	35,990,735	0.81	4771	89.26	18.82	6,541,497	2,800,692
*C. wenyujin*	72,698,023	0.95	5287	98.91	5.38	7,086,543	3,599,640
*C. woodii*	74,469,743	0.94	5184	96.99	6.00	7,001,298	3,531,729
*C. yingdeensis*	70,113,459	0.94	5252	98.26	5.62	7,052,478	3,575,840
*C. zanthorrhiza*	69,596,766	0.94	5269	98.58	5.99	7,069,953	3,565,460

*Note:* SOG represents single‐copy orthologous gene.

### 3.2 Morphological Distinctions of the New Species

**FIGURE 3:**
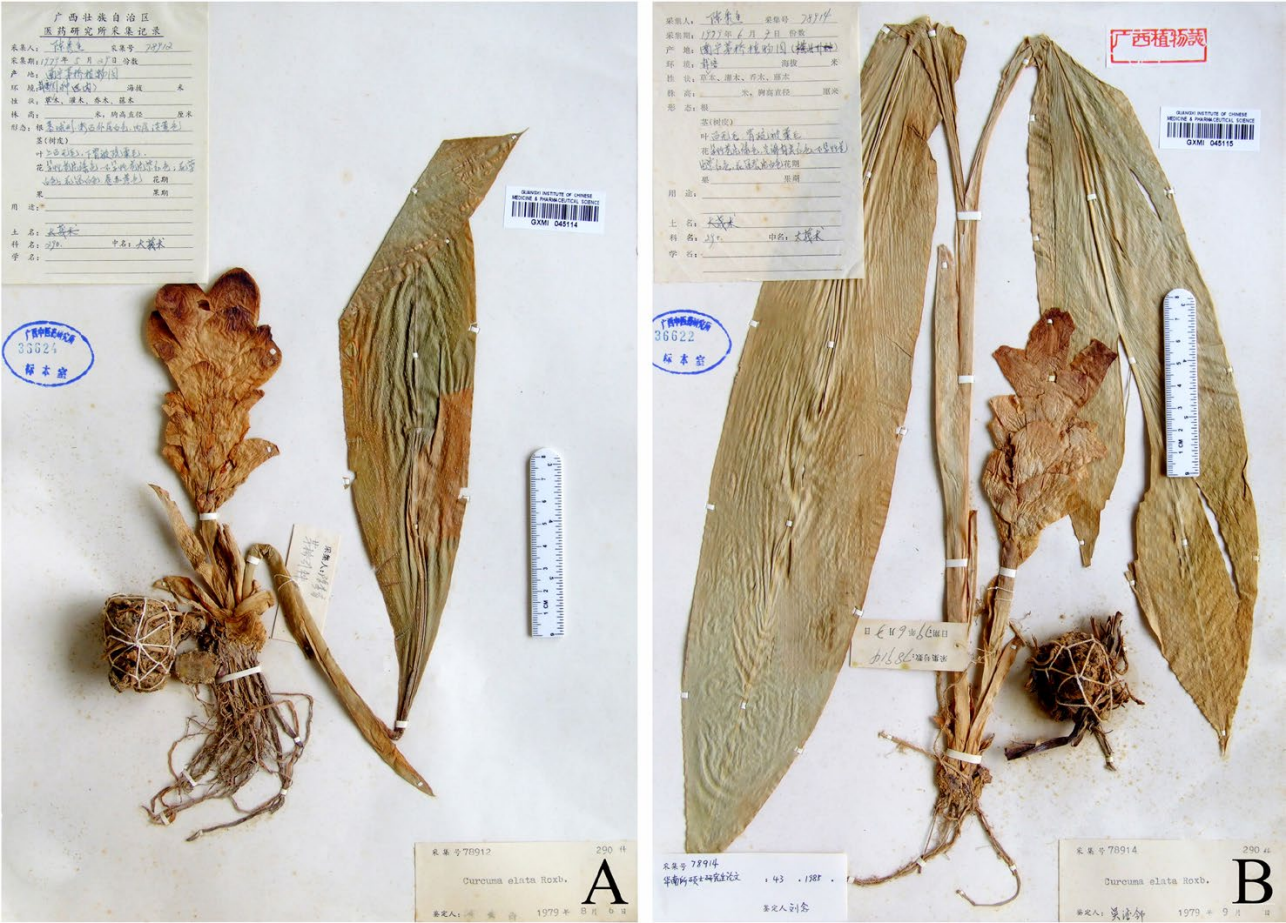
Two specimens misidentified as *C. elata* . (A) *X. X. Chen 78912*. (B) *X. X. Chen 78914*.

**FIGURE 4:**
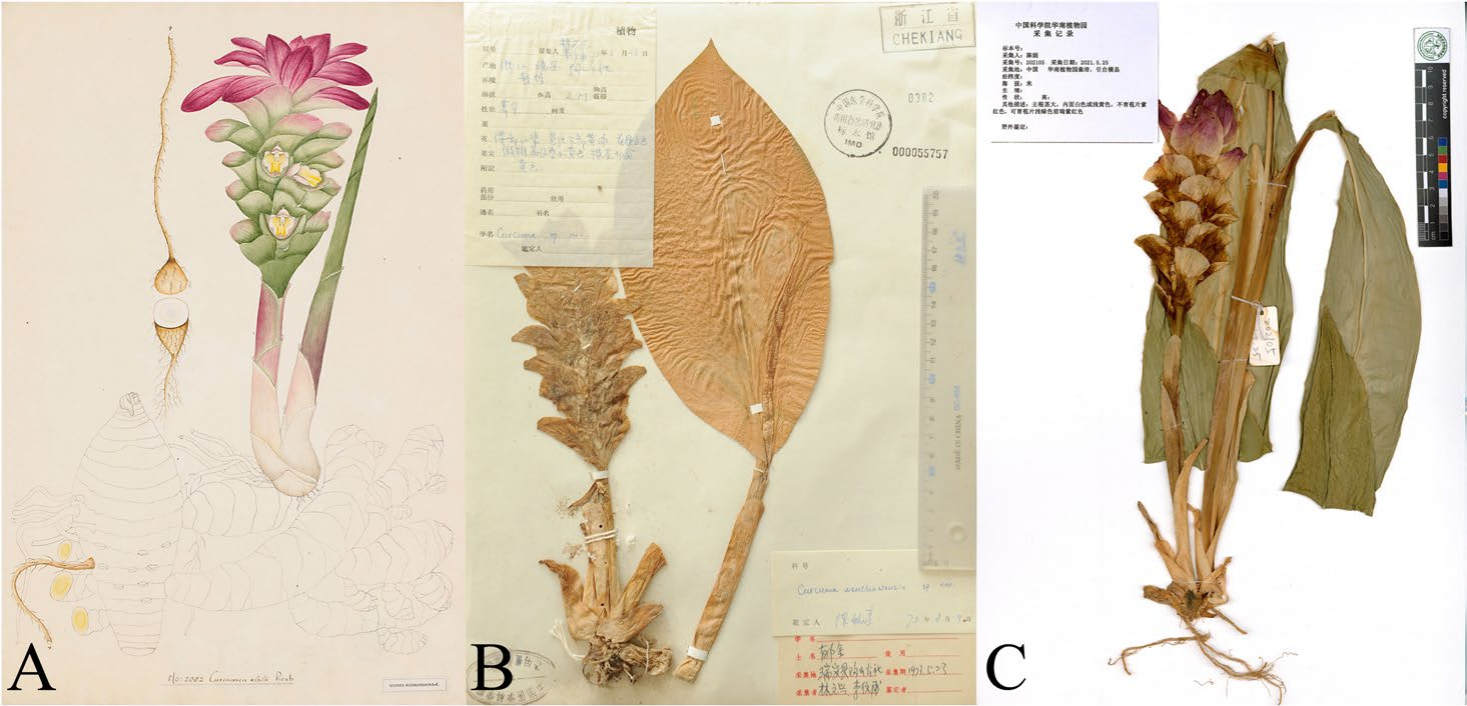
Type of *Curcuma deliniana* N. H. Xia & Juan Chen and its allies. (A) Lectotype of *C. elata* . (B) Holotype of *C. wenyujin*. (C) Holotype of *C. deliniana*.

**FIGURE 5:**
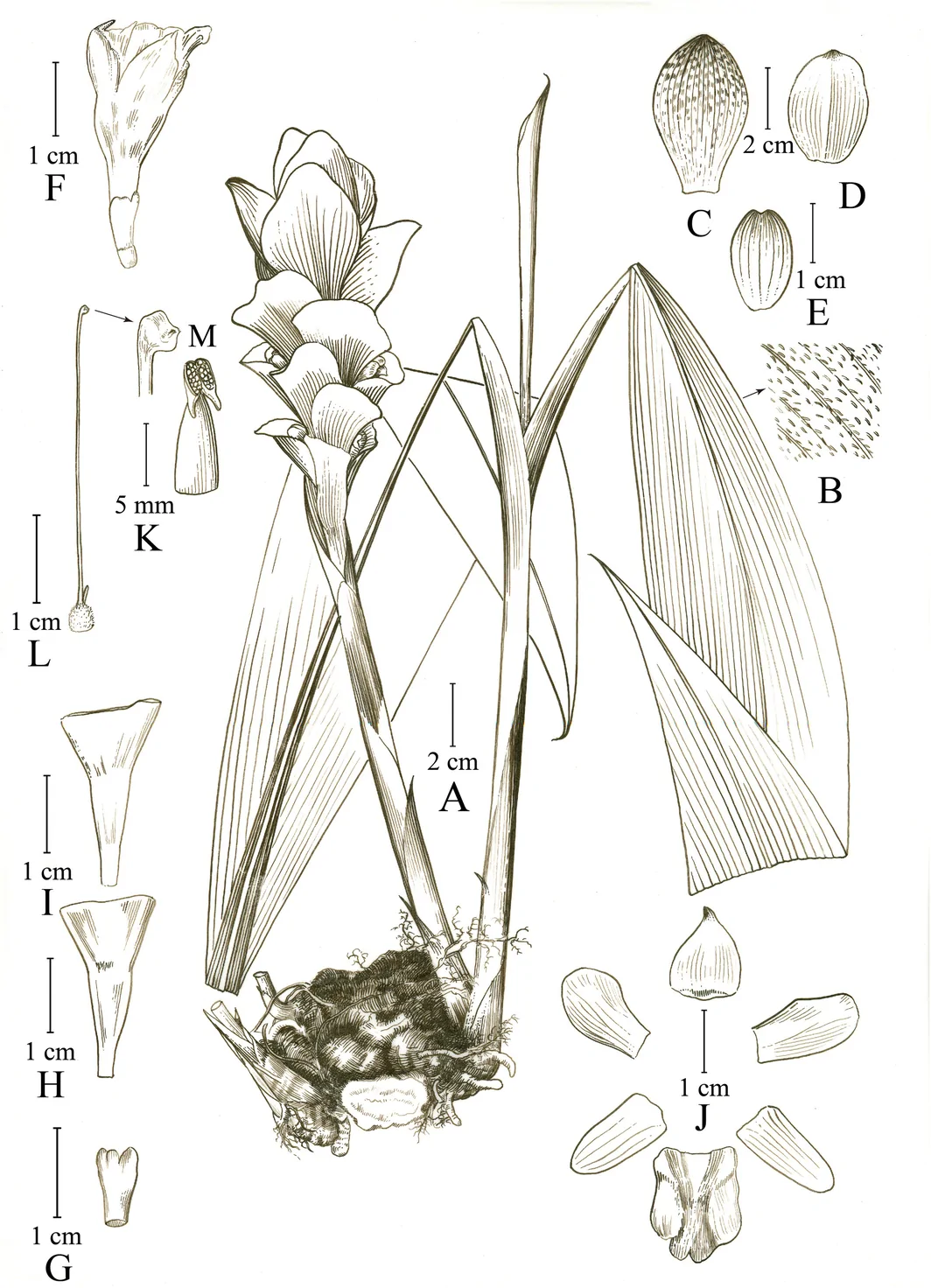
*Curcuma deliniana* N. H. Xia & Juan Chen. (A) Lateral inflorescence with rhizomes and leaf blades. (B) Enlarged view of abaxial leaf surface showing hairs. (C) Coma bract. (D) Fertile bract. (E) Bracteole. (F) Flower. (G) Calyx. (H) Floral tube dissection showing the inner surface. (I) Floral tube dissection showing the outer surface. (J) Dissection of a flower. (K) Stamen. (L) Ovary with epigynous glands, style, and stigma. (M) Enlarged view of a stigma.

**FIGURE 6:**
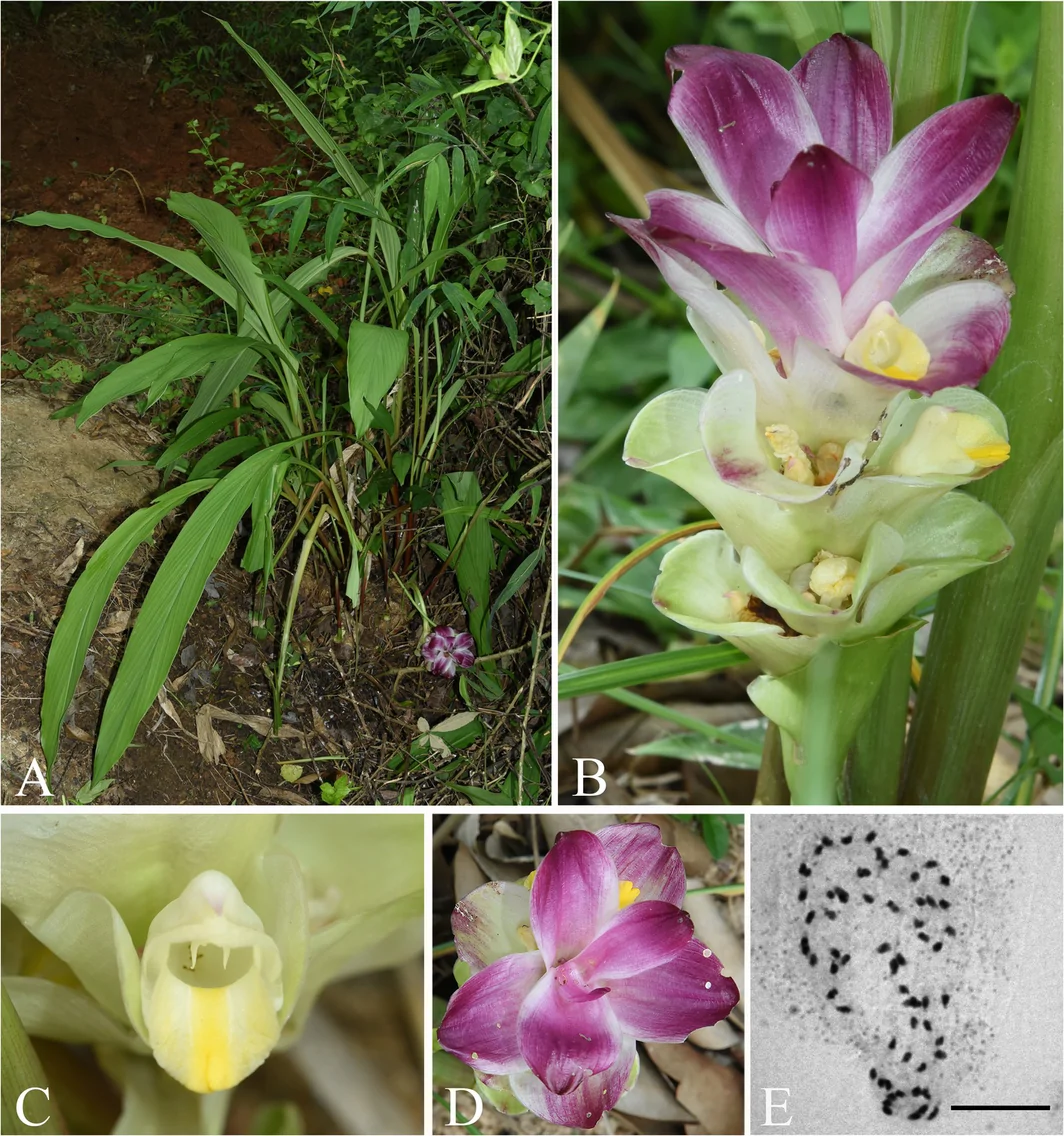
*Curcuma deliniana* N. H. Xia & Juan Chen. (A) Habit in the field. (B) Inflorescence (front view). (C) Flower. (D) Inflorescence (top view). (E) chromosome number 2*n* = 63. Scale bar = 10 μm.

**FIGURE 7:**
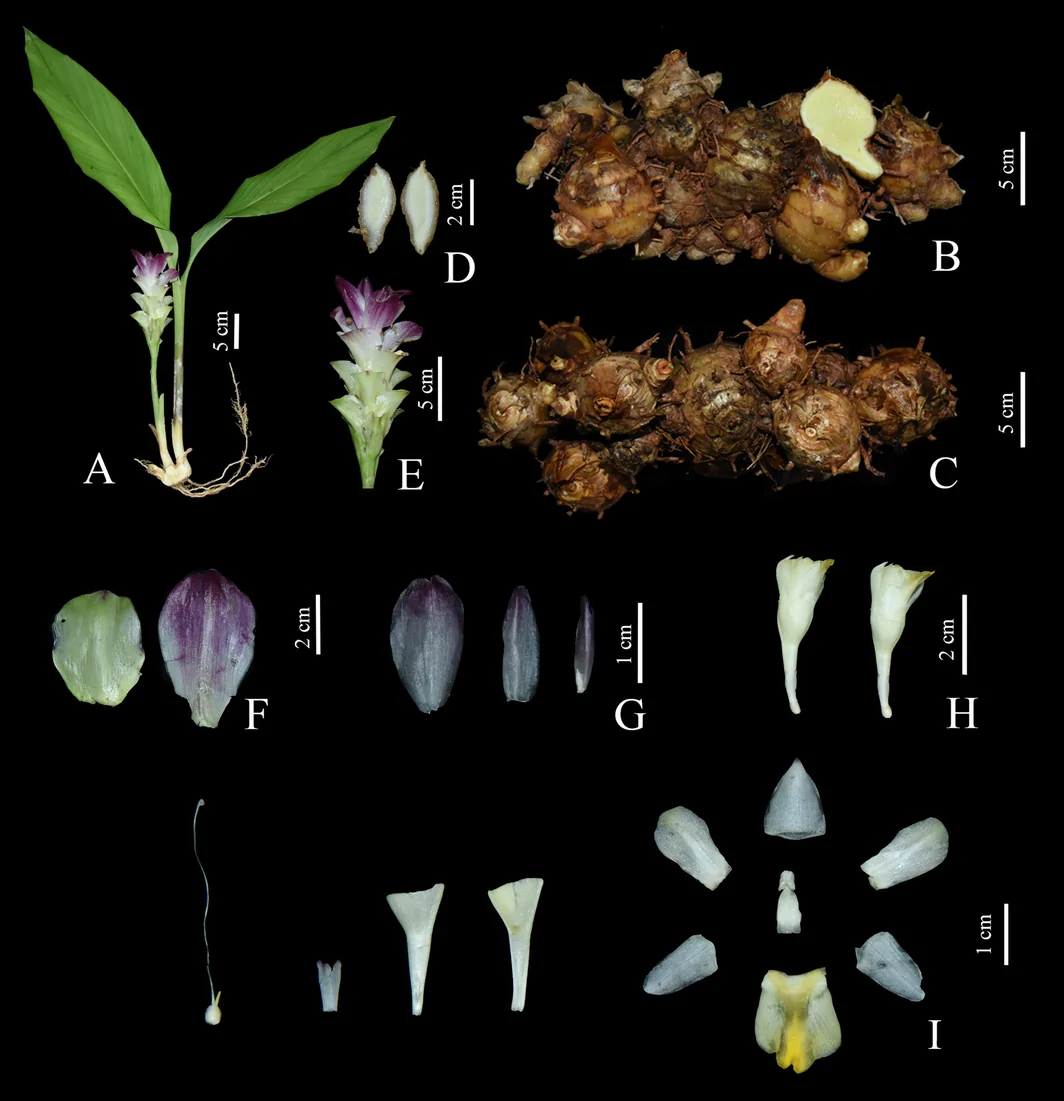
*Curcuma deliniana* N. H. Xia & Juan Chen. (A) The whole plant. (B) Rhizomes (front view and longitudinal section). (C) Rhizomes (top view). (D) Tuber (longitudinal section). (E) Inflorescence. (F) Fertile bract (left) and coma bract (right). (G) Bracteoles. (H) Flowers. (I) Flower dissection: Ovary with epigynous glands, style and stigma followed by calyx, longitudinal dissection of floral tube, lateral staminodes, lateral corolla lobes, dorsal corolla lobe, stamen and labellum.

**FIGURE 8:**
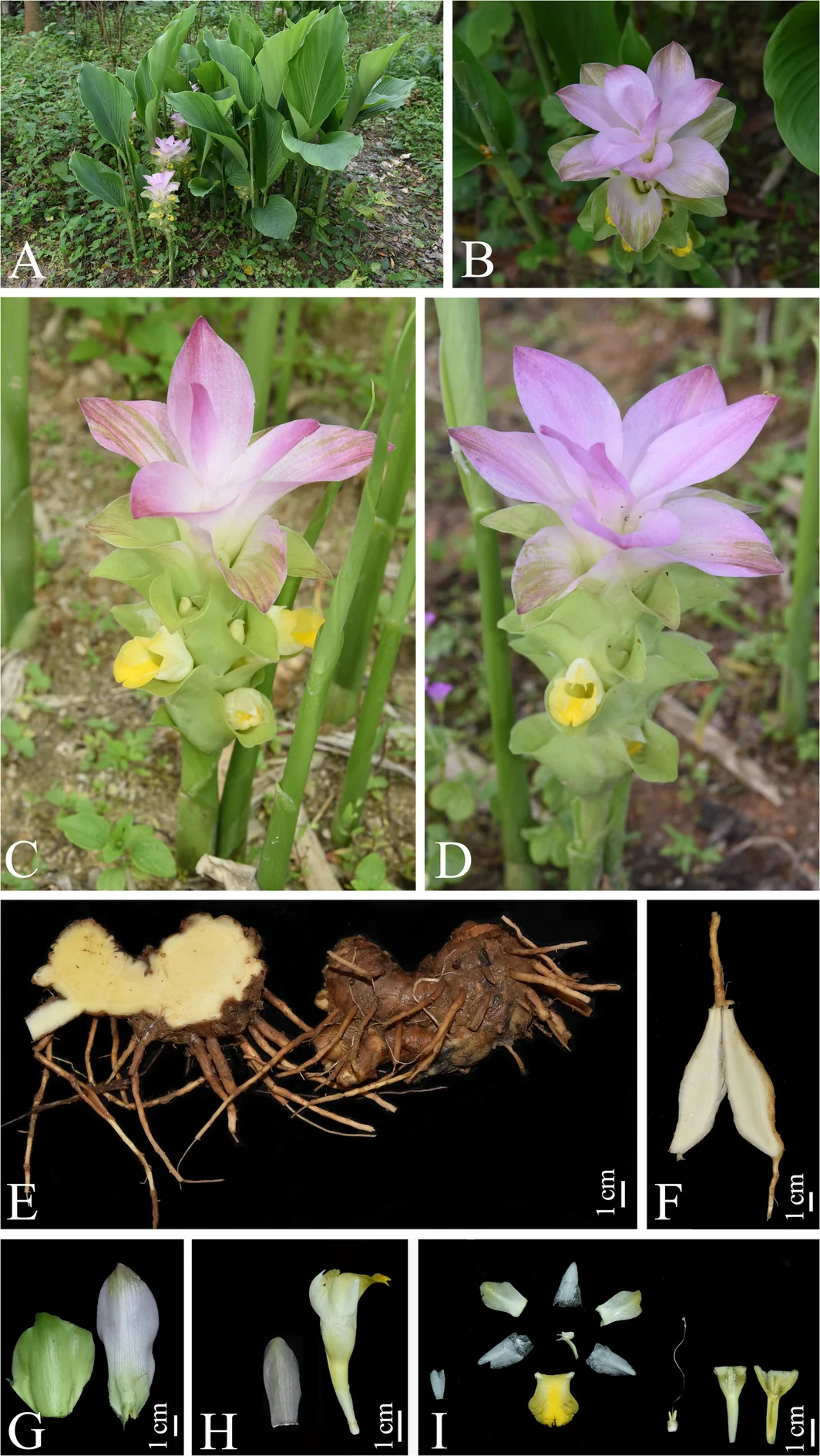
*Curcuma wenyujin* Y. H. Chen & C. Ling. (A) Habit. (B) Inflorescence (top view). (C, D) Inflorescences and flowers. (E) Rhizome. (F) Tuber. (G) Fertile bract (left) and coma bract (right). (H) Bracteole (left) and flower (right). (I) Flower dissection.

The specimens (*X. X. Chen 78914* & *78912*) (Figure 3) mentioned in the Introduction section were collected in 1979 at Guangxi Medical Botanical Garden. They are characterized by small stature (60–80 cm tall); nearly globose, rarely branched rhizomes (4–4.5 × ca. 3.5 cm after drying); narrow leaf blades (31.5–50 × 6.5–11 cm) with glabrous adaxial surfaces and puberulent abaxial surfaces; small, loosely arranged lateral inflorescences; broadly elliptic, purplish red coma bracts; and broadly ovate to rounded fertile bracts. These features match well with Chen's (1980) description of *Curcuma elata* in *Survey of Curcuma in Guangxi*. Meanwhile, these specimens were labeled by X. X. Chen as “大莪术Da E Zhu”, and one bears her handwritten annotation “ *C. elata* ” (Figure 3A). In fact, *Curcuma elata* was originally described by Roxburgh (1820) based on the living materials introduced from Myanmar by Dr. W. Carey to the Botanic Garden at Calcutta, and later was designated “*Curcuma elata*” [icon] in Icons Roxburghianae Ineditae No. 2002 (Figure 4A) at K as its lectotype by Leong‐Škorničková et al. (2010). Liu and Wu (1999) argued that according to the original description, *C. elata* Roxb. is characterized by plants reaching 1.8 m in height and leaves 0.6–1 m long with densely villous abaxial surfaces; however, these morphological traits are distinctly inconsistent with those documented by Chen (1980). They considered that the morphological features described by Chen (1980) fell within the intraspecific variation range of *C. wenyujin* (Figures 4B and 8). Nevertheless, all these specimens differ notably from typical *C. wenyujin* in several key characters. They have narrow leaf blades with sparse pubescence on the abaxial surface, while *C. wenyujin* possesses broad leaves that are entirely glabrous on both sides. In addition, their coma bracts are broadly ovate and purplish red, retaining a deep hue and usually remaining purplish red even in dried herbarium specimens. In contrast, the coma bracts of *C. wenyujin* are elliptic and pink, whose color almost fades completely after drying. All these diagnostic characteristics mentioned above are consistent with the morphological features of this newly described species (Figures 4C and 5, 6, 7).

Specifically, compared to *C. wenyujin*, the new species has a shorter stature (60–110 cm vs. 80–160 cm), rarely branched (vs. much long‐branched) rhizomes, narrower leaf blades (31–60 × 7–13 cm vs. 35–75 × 14–22 cm), reddish brown (vs. green) leaf sheaths, purplish red (vs. pink) coma bracts, broadly ovate to rounded fertile bracts that have an obtuse apex with purple dots (vs. ovate fertile bracts with acute, green tips), smaller (1.5–1.7 × 0.6–1.0 cm vs. 2.5–3.0 × 1.8–2.0 cm) bracteoles and trilobed (vs. bilobed) labellum. The new species also obviously differs from true *C. elata* in its smaller stature (60–110 cm vs. up to 2.4 m), rarely branched rhizomes (vs. much branched rhizomes), smaller leaves (31–60 × 7–13 cm vs. 60–120 × ca. 30 cm), smaller inflorescences, and ovate coma bracts which are much erect, tips non‐reflexed (vs. oblong coma bracts with spreading, slightly reflexed tips).

In the field, the new species grows sympatrically with *C. kwangsiensis* , which is a valuable medicinal herb, specifically the rhizomes (a source of “莪术 E Zhu”) and tubers (“郁金 Yu Jin”), widely cultivated in southwestern China (Guangxi) and officially used in traditional Chinese medicine for treating blood stasis, tumors, and stomach issues. As a seed‐setting species (2*n* = 84), characters of *C. kwangsiensis* such as the purple stripes (presence or absence) on the upper surface of leaf blades, the coma color (white, pink to dark violet) are variable, but the habit, the rhizome branches (rarely branched), the leaf blades indumentum (pubescent on both sides), the fertile bract shape (ovate), the color and shape of the labellum and lateral staminodes are stable within the population observed. However, the chromosome number of the new species was determined to be 2*n* = 63 (Figure 6E), which is a triploid according to the basic chromosome number (*x* = 21) (Chen et al. 2013; Puangpairote et al. 2016). As it is a triploid, it does not produce seeds and characters such as the shape of the rhizome, the indumentum of the leaf blades, the color and shape of the floral parts of this species are stable within the observed materials. Although Chen (1980) reported that the new species was used as a substitute or adulterant of *C. kwangsiensis* in the market, *C. deliniana* is completely different from it in its main rhizome shape, leaf blade shape and indumentum, inflorescence shape and size, coma bract shape, fertile bract shape and color, and corolla lobe color. Detailed comparisons are given in the diagnosis and Table 4.

**TABLE 4:** Morphological comparisons between *Curcuma deliniana* N. H. Xia & Juan Chen and its allies.

Characters	*Curcuma deliniana*	*C. kwangsiensis*	*C. nankunshanensis*	*C. yingdeensis*	*C. wenyujin*
Plant height (cm)	60–110	50–80	80–120	40–70	80–160
Main rhizome					
Shape	Broadly ovoid to globose	Ovoid	Ovoid	Ovoid	Ovoid
Size (cm)	6–8 × 4–6	4–6 × 2.5–3.5	6–9 × 4–6	3.5–10 × 2.5–6	4–5 × 4–5
Rhizome branch	Rarely branched, except a single branch leading to a new shoot	Rarely branched, except a single branch leading to a new shoot	Many	Many	Many
Leafless sheath color	Reddish brown	Green	Reddish brown	Reddish brown	Green
Leaf blade					
Size (cm)	31–60 × 7–13	14–39 × 4–7 (9.5)	55–79 × 7.5–15	ca. 30 × 7–13.5	35–75 × 14–22
Color	Green	Green with purple stripes adaxially	Green with faint purple stripes adaxially when fresh, then disappear	Green with obvious purple stripes adaxially when fresh, then become faint	Green
Indumentum	Adaxially glabrous, abaxially sparsely puberulent or glabrous	Pubescent on both sides	Adaxially glabrous, abaxially pubescent	Pubescent on both sides	Glabrous on both sides
Inflorescence position	Lateral	Lateral and central	Lateral and central	Lateral	Lateral
Number of bracts	16–23	ca. 50	25–33	19–26	23–34
Coma bract	Broadly elliptic	Oblong	Oblong	Oblong	Oblong
Shape					
Size (cm)	4.5–6.0 × 2.2–3.4	4.3–6.8 × 1.3–2.6	5.0–6.0 × 3.0–4.0	4.0–5.5 × 1.6–2.3	6.0–9.0 × 1.2–2.4
Color	Purplish red	Pink to purplish red	Purple with a white base	Pale pink with purple tips	Pink
Apex	Non‐reflexed	Reflexed	Reflexed	Reflexed	Reflexed
Fertile bract					
Shape	Broadly ovate to rounded	Ovate	Ovate‐elliptic	Ovate	Ovate
Color	Creamy or whitish green with purple tips	Green	Green with purple tips	Green with purple tips, usually with purple dots at the adnate portion	Whitish green
Indumentum	Glabrous on both sides	Glabrous on both sides	Pubescent on both sides	Pubescent on both sides	Pubescent on both sides
Fertile bract apex	Obtuse	Obtuse	Acute	Acute	Acute
Bracteole size (cm)	1.5–1.7 × 0.6–1	1.8–2.0 × 1.0–1.2	2.6–2.8 × 1.5–2.0	1.6–1.8 × 0.8–1.0	2.5–3.0 × 1.8–2.0
Calyx color	White with purple tips	White	White	White	White
Corolla color	White	Purple	Purple	Pink	White
Labellum shape	Trilobed, median one 2–3 mm long	Bilobed	Trilobed, median one ca. 5 mm long	Bilobed	Bilobed
Chromosome number (2*n*)	63	84	84	63	63

The new species is also related to *C. nankunshanensis*, but can be distinguished from it by rarely branched main rhizomes (vs. long branched rhizomes), green leaf blades (vs. green leaf blades with faint purple stripes adaxially), purplish red (vs. purple with a white base) coma bracts, much erect, non‐reflexed (vs. reflexed) at tips, whitish green fertile bracts with obtuse tips (vs. green fertile bracts with acute tips), smaller (1.5–1.7 × 0.6–1 cm vs. 2.6–2.8 × 1.5–2.0 cm) bracteoles and white (vs. purple) corolla lobes, and tri‐lobed labellum with a shorter median lobe (2–3 mm long vs. 5–6 mm long). Additionally, the flowering times of the two species are also distinct, with *C. nankunshanensis* flowering twice per year (early April to June and August to September), while the new species flowering once, from May to July.

It somewhat resembles *C. yingdeensis* but readily differs from it in several key characteristics: its rarely branched main rhizomes (vs. long‐branched rhizomes), green leaf blades, adaxially glabrous, abaxially sparsely puberulent or glabrous (vs. green leaf blades with obvious purple stripes, pubescent on both sides), more broadly elliptic and purplish red (vs. oblong, pink with purple tips) coma bracts, much erect, non‐reflexed (vs. spreading, reflexed) at tips, broadly ovate to rounded and whitish green fertile bracts with obtuse tips (vs. ovate and pale green fertile bracts with acute tips), white (vs. pink) corolla lobes, and trilobed (vs. bilobed) labellum. Their main differences are presented in Table 4.

## 4 Taxonomic Treatments

### 4.1 *Curcuma deliniana* Juan Chen & N. H. Xia sp. *Nov.* (Figures 4C and 5, 6, 7)

= *Curcuma elata* auct. non Roxb.: Chen in Guihaia 2: 5. 1980; Chen in Acta Pharmaceutica sinica 16(5): 388. 1981.

#### 4.1.1 Type

CHINA. Guangdong: Guangzhou, 25 May 2021, *J. Chen 202105* (holotype: IBSC0938854, isotypes: KUN, PE). Materials originally introduced from Hengzhou, Guangxi, were cultivated at South China Botanical Garden (cultivation accession no. 060912), from which specimens were collected.

#### 4.1.2 Diagnosis

It is similar to *C. kwangsiensis* but differs in its larger, nearly globose (6–8 × 4–6 cm vs. 4–6 × 2.5–3.5 cm, ovoid) main rhizomes; green leaf blades, adaxially glabrous, abaxially sparsely puberulent or glabrous, much spreading (vs. green leaf blades with purple stripes, pubescent on both sides, much erect); inflorescence with fewer, loosely arranged bracts (16–23 vs. ca. 50, densely arranged); more broadly elliptic coma bracts, non‐reflexed at tips (vs. oblong coma bracts, reflexed at tips); nearly rounded, whitish green fertile bracts with purple tips (vs. ovate, green fertile bracts); white (vs. purple) corolla lobes; and chromosome number 2*n* = 63 (vs. 2*n* = 84).

#### 4.1.3 Description

Rhizomatous herbs, 60–110 cm tall. *Main rhizome* 6–8 × 4–6 cm, broadly ovoid to globose, aromatic, bitter, dark brown externally, pale yellow to creamy internally, readily oxidized after cutting, rarely branched, except a single branch leading to a new shoot; root tubers many, 3.5–3.8 × 1.6–2.0 cm, obovate, brown to white externally, white internally. *Leafy shoots* usually with 2–3 leaves at anthesis; pseudostems slender, composed of 2–3 leafless sheaths and 2–3 leafy sheaths; leafless sheaths reddish brown, glabrous; upper part of leafy sheaths green, lower part brown, glabrous; ligules ca. 2 mm in height, bilobed, hyaline, ciliate on margin; petioles 3–8 cm long (petiole of first leaf shortest, innermost ones longer), green, glabrous; leaf blade 31–60 × 7–13 cm (measured at anthesis), narrowly elliptic, base acuminate, slightly oblique, apex attenuate with uppermost tip pubescent, margin entire and glabrous, pale green, adaxially glabrous, abaxially sparsely puberulent or glabrous. *Inflorescence* lateral; spike 11–18 × 5–10 cm, consisting of 16–23 bracts; peduncle erect, sometimes curved, 12.0–21.5 cm long, green, glabrous; sheaths green, glabrous; coma bracts 4.5–6.0 × 2.2–3.4 cm, broadly elliptic, spreading, non‐reflexed, base involuted, apex obtuse and mucronate, purplish red, glabrous to sparsely puberulent on both sides; fertile bracts 3.2–4.1 × 2.0–3.2 cm, broadly ovate to rounded, mostly erect, whitish green with purple tip, connate to 1/2 above base, base involuted, apex obtuse, glabrous on both sides, loosely arranged; cincinni with 3–5 flowers at base of inflorescence, the number gradually decreasing upwards; bracteoles one per flower, the outer largest one 1.5–1.7 × 0.6–1.0 cm, the remaining ones gradually decrease in size, elliptic, apex acute, white with purple tip, glabrous on both sides. *Flowers* 3.2–4.2 cm long, not exserted from bracts; calyx tubular, 8–9 mm long, inconspicuously tridentate, white with purple tip, glabrous externally; floral tube 1.8–2.4 cm long, narrowly cylindrical at base, funnel‐shaped at apex, white, glabrous externally, internally white and glabrous in basal 1/2, densely villous at throat, distally creamy white, sparsely puberulous; lateral corolla lobes 1.2–1.5 × 0.6–0.8 cm, elliptic, apex obtuse and slightly concave, white, glabrous on both sides; dorsal lobe 1.4–1.5 × 0.8–1.0 cm, elliptic and concave, white, glabrous on both sides, apex mucronate, mucro 2–3 mm long, white, glabrous; lateral staminodes 1.5–1.7 × 0.7–0.9 cm, oblanceolate, yellowish white, glabrous on both sides; labellum 1.5–1.8 × 1.4–1.5 cm, broadly obovate, obscurely trilobed, with a 2–3 mm long incision at center, yellowish white, two yellow bands at center, glabrous on both sides; stamen 1.0–1.2 cm long; filament ca. 7 mm long, broad, flat, white; anther 3–5 mm long, white; spur short, 2–3 mm long, white, narrowly triangular with sharp tips pointing downwards; anther crest absent; epigynous glands 2, 2–3 mm long, cylindrical with pointed apex, white cream; style white, glabrous; stigma ca. 1 mm wide, capitate, white, ostiole ciliate; ovary 2–3 mm long, white, pubescent externally. *Fruits* and seeds unseen.

#### 4.1.4 Phenology

Flowering from May to July.

#### 4.1.5 Etymology

The specific epithet is to commemorate Prof. Wu De‐Lin for his remarkable contribution to the taxonomy of Chinese gingers. The Chinese vernacular name is “德邻郁金” [dé lín yù jīn].

#### 4.1.6 Distribution, Habitat and Conservation Status

Two locations are documented in Hengzhou and Yulin of Guangxi, China. Among them, occurrence in Yulin is supported by existing herbarium specimens. Field surveys at the type locality in Hengzhou revealed a small population growing in a damp valley in unprotected areas. Additionally, the species is subject to sporadic exploitation for commercial medicinal purposes; this harvesting pressure is inferred to drive a continuing decline in habitat quality and the number of mature individuals within populations outside protected areas. Calculations following Bachman et al. (2011) estimated its Extent of Occurrence (EOO) as 0 km^2^ and its Area of Occupancy (AOO) as 8 km^2^. The EOO = 0 km^2^ arises because the two known localities cannot form a meaningful minimum convex polygon under the implemented calculation framework. Although these EOO/AOO values satisfy the quantitative B1/B2 thresholds for Critically Endangered, we retain a provisional Endangered rating because total population size and full distribution range remain incomplete. Given that the species is known from two localities and suffers from persistent threats across both sites, we provisionally assess this species as Endangered (EN B1ab(iii, v), B2ab(iii, v)) following IUCN Red List Categories and Criteria (IUCN 2012; IUCN Standards and Petitions Committee 2024). If future surveys confirm no further populations exist, the species may qualify for Critically Endangered; conversely, discovery of further populations may warrant a downgrade in category.

#### 4.1.7 Additional Specimens Examined

CHINA. Guangxi: Yulin, 26 May 1959, *Y. K. Li 404417* (IBK), *Y. K. Li 404437* (IBK, only inflorescence included); Nanning, cultivated at Guangxi Medical Botanical Garden, 11 July 1984, *C. C. Huang 13855* (GXMG), 29 May 1979, *X. X. Chen 78912* (GXMI), 7 June 1979, *X. X. Chen 78914* (GXMI, IBSC), 16 May 2008, *J. Chen 0807* (IBSC), 23 May 2014, *Y. Tong 14052201* (IBSC).

### 4.2 ***Curcuma wenyujin*** Y. H. Chen & C. Ling in Acta Pharm. Sinica 16 (5): 387. 1981

#### 4.2.1 Type

CHINA. Zhejiang: Ruian, Taoshan, 23 May 1973, *W. X. Lin & X. Z. Li s.n*. (holotype: IMD000055757!; isotypes: KUN!, ZJ!). (Figures 4B and 8).

#### 4.2.2 Phenology

Flowering from April to June.

#### 4.2.3 Chinese Vernacular Name

The Chinese vernacular name is “温郁金” [wēn yù jīn].

#### 4.2.4 Distribution, Habitat and Conservation Status

This species occurs in Fujian, Guangxi, Guangdong, and Zhejiang and has been cultivated in Zhejiang as a medicinal herb for a long time. Given its wide distribution and an estimated population exceeding 10,000 mature individuals, the species should be classified as Least Concern (LC) according to the Guidelines for Using the IUCN Red List Categories and Criteria (IUCN Standards and Petitions Committee 2024).

#### 4.2.5 Additional Specimens Examined

CHINA. Fujian: Wuping, 12 June 1959, *anonymous 3679* (FJIDC). Guangxi: Linshan, *J. Chen 201415 & 201416* (IBSC); Nanning, cultivated at Guangxi Medical Botanical Garden, 6 May 1976, *X. X. Chen 78896* (GXMI), 17 May 1979, *X. X. Chen & H. H. Feng 78911* (GXMI), 13 June 1985, *C. C. Huang 10058* (GXMG), *J. Chen 201403* (IBSC). Guangdong: Guangzhou, cultivated at South China Botanical Garden, 9 May 2017, *J. Chen 17050903* (IBSC); Lechang, 18 May 1934, *S. B. Guo 80623* (IBSC); Yingde, 8 May 2017, *N. H. Xia et al. 17050802* (IBSC), 17 May 2017, *J. Chen et al. 17051701* (IBSC). Zhejiang: Ruian, Mayu, 29 May 1972, *anonymous s.n*. (IBK); ibid., Taoshan, 7 June 1976, *C. Ling 1606 & 1607* (PE, IBSC), 28 May 1981, *C. Ling & F. G. Lai 3003* (ZDC), 5 June 1995, *X. S. Zheng s.n*. (NAS), 13 June 2013, *J. Chen 201301 & 201302* (IBSC).

#### 4.2.6 Notes

In the protologue of *Curcuma wenyujin*, Y. H. Chen (1981) stated that the description was based on *W. X. Lin & X. Z. Li s.n*., but did not indicate where the type specimen was preserved. After an extensive investigation, a single sheet matching the description and the collection information has been located at IMD where Chen worked (with the help of Dr. Hai‐Tao Li of IMDY) (Figure 4B), which is regarded here as the holotype. Additionally, two other sheets of *W. X. Lin & X. Z. Li s.n*. preserved at KUN and ZDC respectively have also been located, which well match the description and collection information, and thus are recognized as isotypes of *C. wenyujin*.

## Author Contributions

**Juan Chen:** conceptualization (equal), data curation (equal), funding acquisition (lead), investigation (lead), methodology (equal), writing – original draft (lead), writing – review and editing (lead). **Hui‐Hong Wang:** formal analysis (equal), software (equal), writing – original draft (equal), writing – review and editing (equal). **En‐Wei Tian:** methodology (lead), project administration (equal), writing – review and editing (equal). **Nian‐He Xia:** conceptualization (lead), project administration (lead), writing – review and editing (equal).

## Funding

This study was supported by the National Natural Science Foundation of China (Grant No. 32070223), the National Key Research and Development Program of China (Grant No. 2024YFF1307400), and the Guangdong Flagship Project of Basic and Applied Basic Research (Grant No. 2023B0303050001).

## Conflicts of Interest

The authors declare no conflicts of interest.

## Data Availability

Sequence alignments, 973 individual gene trees, and a multi‐tree file containing all gene trees used for coalescence‐based species tree inference are available at figshare (https://figshare.com/s/130592eab87973da6330). GenBank accession numbers of the plastid genomes are provided in Table 1.
